# Histone regulator KAT2A acts as a potential biomarker related to tumor microenvironment and prognosis of diffuse large B cell lymphoma

**DOI:** 10.1186/s12885-023-11401-4

**Published:** 2023-10-03

**Authors:** Zhuoya Yu, Mengfei Ding, Yiqing Cai, Tiange Lu, Xiaomin Chen, Xiangxiang Zhou, Xin Wang

**Affiliations:** 1 Department of Hematology, Shandong Provincial Hospital, Shandong University, No.324, Jingwu Road, Jinan, 250021 Shandong China; 2 Department of Hematology, Shandong Provincial Hospital Affiliated to Shandong First Medical University, Jinan, 250021 Shandong China; 3 Branch of National Clinical Research Center for Hematologic Diseases, Jinan, 250021 Shandong China; 4 National Clinical Research Center for Hematologic Diseases, the First Affiliated Hospital of Soochow University, Suzhou, 251006 China

**Keywords:** DLBCL, Histone acetylation, Tumor microenvironment, KAT2A, Immunotherapy

## Abstract

**Background:**

Recent studies have indicated that epigenetic alterations contribute significantly to lymphoma pathogenesis. A type of epigenetic regulation known as histone acetylation plays a crucial role in transcriptional regulation in eukaryotic cells. Specifically, a significant effect of histone acetylation modifications on the abnormal progression and microenvironment of diffuse large B-cell lymphoma (DLBCL) has been observed.

**Methods:**

To provide insight into the significance of histone acetylation-related genes, we developed a HAscore model for analyzing histone acetylation patterns in DLBCL samples. Furthermore, KAT2A, a regulator of histone acetylation, was knocked down in DLBCL cell lines to investigate its role in proliferation, cell cycle, and apoptosis.

**Results:**

The HAscore model has been demonstrated to provide insight into the significance of these patterns, showing that patients with a low HAscore have distinct tumor immune microenvironments and poorer prognoses. Besides, KAT2A was identified as a potential biomarker related to immune infiltration and malignant pathways in DLBCL.

**Conclusion:**

According to these findings, it is evident that the histone acetylation pattern score model is helpful in describing the immune status of DLBCL and that KAT2A may be used as a biomarker for its treatment.

**Supplementary Information:**

The online version contains supplementary material available at 10.1186/s12885-023-11401-4.

## Background

Diffuse large B-cell lymphoma (DLBCL) is a highly aggressive form of non-Hodgkin’s lymphoma (NHL), originating from B-lineage lymphocytes, accounting for 30–40% of cases [1–3]. Moreover, this lymphoid neoplasm has highly variable gene expression profiles and genetic alterations [4, 5]. The most common up-front treatment is R-CHOP [6]. However, due to its heterogeneity, 60% of patients are curable with combination therapy and the remainders still succumb to the disease [7]. Unfortunately, those who develop the disease refractory to up-front treatment or relapse after remission have a poor prognosis [8–10]. To understand the underlying mechanisms of DLBCL progression, more studies are necessary. As a result, stratifying DLBCL patients and developing predictive models can provide more precise molecular subtyping and, therefore, more customized treatment.

Epigenetic alterations are directly linked to lymphoma pathogenesis. As one of these post-translational modifications, histone acetylation has been extensively studied [11]. Post-translational modifications of histones regulate transcription and DNA repair and are linked to the stable maintenance of repressive chromatin [12]. The N-terminal tail of histones contains lysine residues that are tightly regulated by acetylation regulators. Acetylation reactions are catalyzed by histone acetyltransferases (HATs), while deacetylation reactions are catalyzed by histone deacetylases (HDACs) [13]. Besides, other regulators recognize modified histones and play a role in acetylation. The three types of regulators are regarded as “writers,“ “readers,“ and “erasers.“ Increasing evidence suggests that the tumor microenvironment (TME) plays a critical role in malignancy development and maintenance. The process is accomplished through sustained proliferation and immune evasion [14, 15]. As a result of several signal molecules being activated and inhibited, histone acetylation has recently been linked to cancer and TME. In various types of human malignancies, aberrant expression of HDACs has been reported [16, 17]. It has also been reported that many HDAC inhibitors are effective against several hematologic and solid malignancies [18, 19]. The HAT paralogs p300 and CBP are involved in many vital cellular processes and have critical roles in several pathological conditions, including cancer [20–22]. Current studies indicated that somatic mutations affecting CREBBP and EP300 are a hallmark of DLBCL [23]. Multiple studies have also shown that histone deacetylase inhibitors (HDACi) can improve the abilities of the immune system to eradicate tumor cells by changing the TME through various mechanisms [24, 25].

Collectively, histone acetylation is critical in regulating TME. Targeting histone acetylation modulators can disrupt the resistance to cancer immunotherapy. Recent research has concentrated on individual histone acetylation modulators and their impact on cancer treatment and prognosis. We retrospectively collected transcription information from public databases to better understand how histone acetylation regulators impact the immune system. We investigated histone acetylation regulatory variables and infiltration of immune cells, as well as the value of HAscore in targeted DLBCL immunotherapy.

## Materials and methods

### Dataset acquisition and clinical samples

The Gene Expression Omnibus (GEO) and the Cancer Genome Atlas (TCGA) databases provided gene expression data and comprehensive clinical annotations. Transcriptome data was derived from fragments per kilobase million and converted into transcripts per million (TPM) and then transformed into log2 (TPM + 1) values for further analysis. The UCSC Xena database also provided genomic mutations (including somatic mutations and copy number variations (CNV). This study focused on the DLBCL cohorts (GSE10846 and GSE31312) as well as the TCGA-DLBC. The R package limma “normalizeBetweenArrays” package was used for normalization after gene symbol conversion (R version: 4.1.2; Bioconductor version: 3.13). A method in “sva” package called “ComBat” was utilized to adjust for batch effects caused by non-biotechnical bias [26, 27].

### Cell lines

Human DLBCL cell lines OCI-LY1, OCI-LY3, OCI-LY8, OCI-LY10, and U2932 were cultured in IMDM (Gibco, MD, USA), supplemented with 10% FBS (Gibco). The cells were maintained under optimal conditions, with a temperature of 37 °C and a humid atmosphere containing 5% CO2. Peripheral blood mononuclear cells and serum were isolated from healthy volunteers.

### Lentiviral generation and cell transfection

The stable knockdown of KAT2A was encoded by cloning the shRNAs into lentiviral vectors (Beijing Syngentech Co., Ltd., China). The manufacturer’s instructions were followed for lentivirus infection. To select the stably transfected cells, the medium was supplemented with puromycin (2.0 g/ml, Sigma-Aldrich, USA). A 72-hour timeframe was used for the collection and analysis of cells.

### qRT-PCR

Total RNA was extracted using RNAiso Plus (TaKaRa, Dalian, China) following the manufacturer’s protocol. Reverse transcription reactions were undertaken using reverse transcription reagents (Vazyme). Expression levels of specific genes were then measured using qRT-PCR on LightCycler 480II system (Roche, Basel, Switzerland). Normalized results were determined based on GAPDH expression.

The KAT2A primer sequence was as follows:

KAT2A: forward 5-CCCGCTACGAAACCACTCAT-3, reverse 5-GCATGGACAGGAATTTGGGGA-3.

### Cell proliferation assay

The cells were added to 96-well plates at a density of 1 × 10^4^ per well. The proliferation of cells was determined after 24, 48, and 72 h of exposure to CCK-8 (Dojindo Laboratories, Kumamoto, Japan). After incubation for four hours, the OD was measured at 450 nm.

### Western blot

The Western blot analysis was performed as described previously [10]. The primary antibody used in this assay was KAT2A (66575-1-Ig, Proteintech). GAPDH was used as an internal reference.

### Flow cytometry

Cell cycle and apoptosis were determined by Navios flow cytometer. Cells were stained for 15 min and monitored for cell cycle. Annexin V-PE/7-aminoactinomycin (7AAD) apoptosis detection assays (BD Biosciences) were used to detect apoptosis.

### Consensus clustering analysis

38 recognized histone acetylation regulators were isolated from the published literature [28–30]. An unsupervised clustering algorithm of 884 DLBCL samples was performed based on their expression levels for 38 histone acetylation regulators. The patients were clustered using the R package “consensusClusterplus”, and the classification was performed 1,000 times to ensure stability [31]. Principal Component Analysis (PCA) was employed to illustrate the clustering conditions of given samples with tumors by dimension reduction.

### Gene set variation analysis (GSVA)

We used the “GSVA” R package to analyze differences in histone acetylation modification patterns between biological processes. The gene sets “c2.cp.kegg.v7.5.1.symbols” were accessed from MSigDB. Genes related to histone acetylation modifications were functionally annotated with a threshold of FDR < 0.05.

### Identification of DEGs in histone acetylation modification characteristics

Patients were divided into three groups by their expression of histone acetylation regulators. An analysis of differentially expressed genes (DEGs) exhibiting various characteristics among histone acetylation patterns was conducted using the limma R package [32]. Accordingly, the DEGs were selected based on |logFC|> 1 and adjusted *P* values < 0.001. Kyoto Encyclopedia of Genes and Genomes (KEGG) [33–35] pathway analysis and Gene Ontology (GO) biological were processed using the R package “clusterProfiler” and “org.Hs.eg.db”.

### Estimation of tumor environment cell infiltration

Adapted from Charoentong’s study [36], the gene set was used to define immune cell types. A fraction of the immune cells infiltrating each sample was determined based on their relative abundance. Based on the single-sample gene set enrichment analysis (ssGSEA) algorithm, ESTIMATE was calculated by the R package “ESTIMATE” based on immune cell and stromal cell-specific gene expression levels to calculate a score reflecting the level of immune cell and stromal cell infiltration. Boxplots of ESTIMATE score, immune score, and stromal score for different risk groups were demonstrated using the R package “ggpubr”.

### Evaluation and generation of HAscore

There has been an effort to develop a method for quantifying the pattern of histone acetylation modifications by developing a scoring approach (HAscore). The overlapping DEGs from different HA clusters were first accessed to divide patients into several clusters via unsupervised clustering. Following a univariate Cox regression model, prognostic-related DEGs were selected to construct HA gene signatures. Following determining the prognostic value of gene signature scores, a method similar to the Genome Grading Index [37] was applied to define the HAscore for each patient: HAscore = ∑(PC1i + PC2i), where i indicates the expression value of each histone acetylation regulator.

### Statistical analysis

Patients’ survival was analyzed by the Kaplan-Meier analysis, and overall survival (OS) between subgroups by the log-rank test was determined. Using the “surv-cutpoint” function in the “survminer” R software package, the optimal cut-off point was determined, resulting in the classification of patients into high- and low-HAscore subgroups, as well as high- and low-KAT2A expression subgroups. The difference between three or more groups was examined using one-way ANOVA and Kruskal-Wallis tests [38]. *p* values were analyzed using two-sided statistical tests in all statistical analyses, and *p* < 0.05 was considered statistically significant. At least three independent experiments were conducted, and the mean and standard deviation (SD) were used in describing the experimental data. R and GraphPad Prism (version 8.0) were utilized to conduct all the statistical analyses.

## Results

### Genetic variants of DLBCL histone acetylation regulators

**Fig. 1:**
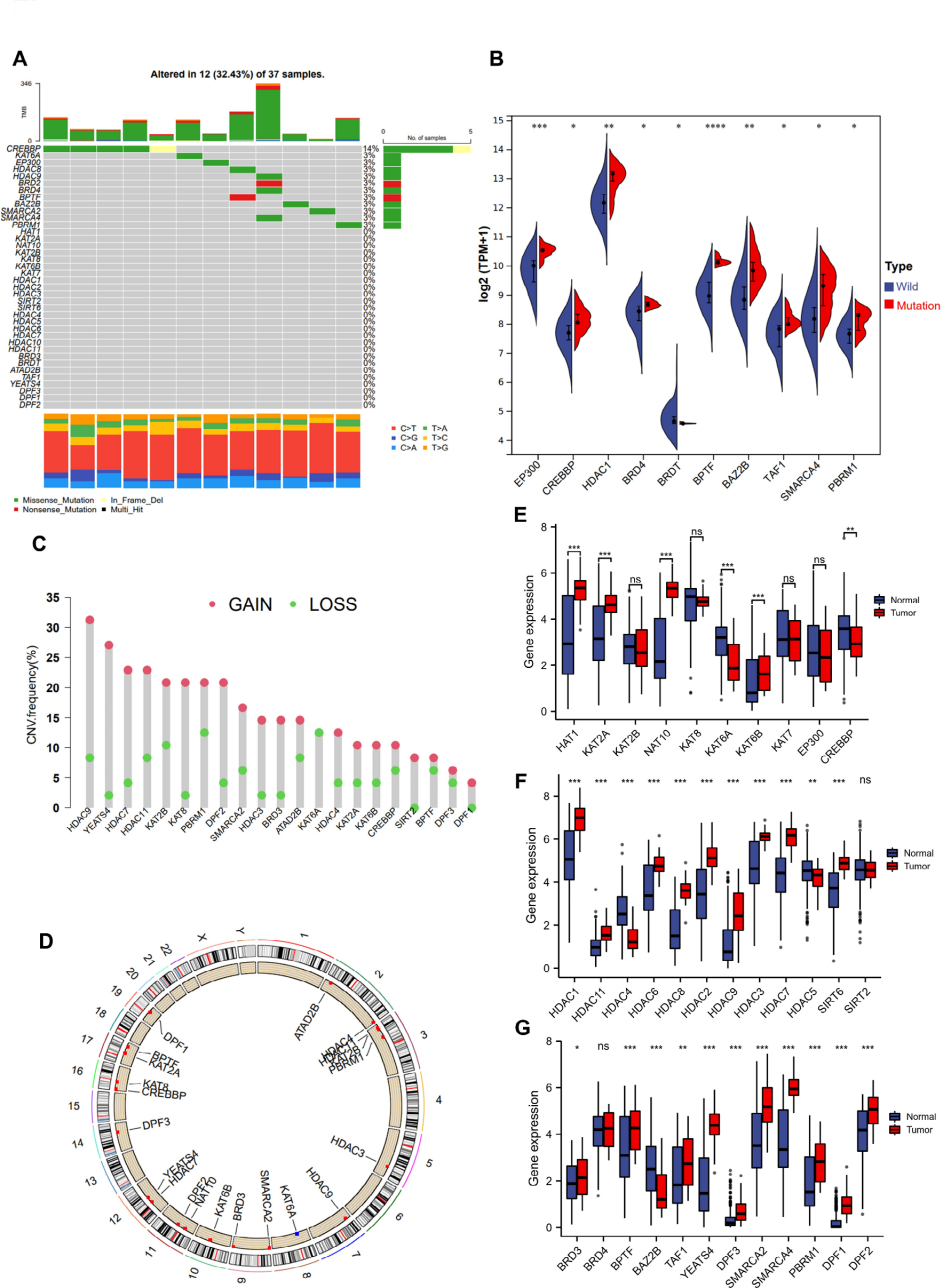
Molecular characterization and expression variation landscape of HA regulators in DLBCL. **(A)**: Mutation frequencies of 38 HA regulators in 37 DLBCL patients. **(B)**: HA regulator expression in mutation and wild groups. **(C)**: The CNV frequency of HA regulators in DLBCL. **(D)**: The position of the histone acetylation regulators CNV on 23 chromosomes. **(E-G)**: 38 HA regulator expression in normal tissue (blue) and tumor tissue (red). The top and bottom of the boxes indicate the interquartile range of values. The asterisk represents the statistical *p*-value (*p < 0.05, ** *p* < 0.01, *** *p* < 0.001).

A systematic review of histone acetylation publications identified 43 histone acetylation regulatory genes, including 10 “writers”, 18 “erasers”, and 15 “readers” (Table S1). The rate of somatic mutations of 43 histone acetylation regulators was assessed to determine the genetic alterations in DLBCL. As evidenced by the TCGA database, 12 of the analyzed samples had mutations in histone acetylation regulators, with a frequency of 32.43% (Fig. 1A). The study results showed that CREBBP had the highest mutation frequency, followed by KAT6A, EP300, HDAC8, HDAC9, BRD4, BRD2, PBRM1, SMARCA2, BAZ2B, and SMARCA4. The remaining genes had no mutations in the DLBCL samples. In terms of the mutation status of CREBBP, DLBCL samples were grouped into two categories: mutation and wild group. Several genes were found to be overexpressed in the mutation group (Fig. 1B). Regarding CNVs, HDAC9, YEATS4, HDAC7, HDAC11, and KAT2B exhibited high amplification rates, whereas KAT6A exhibited copy number loss (Fig. 1C). Chromosome-wide copy number alterations of HA regulators have been identified (Fig. 1D). Subsequently, we investigated the expression levels of histone acetylation regulators in both normal and DLBCL tissues in “writers”, “erasers”, and “readers”, respectively, to investigate the differences in the expression of these regulators in the two groups of sample (Fig. 1E-G). Most genes were upregulated in DLBCL samples compared to normal samples, except for HDAC5, DPF3, and SMARCA2. There is a high degree of heterogeneity in histone acetylation regulators, suggesting that aberrant expression patterns of HA regulators may contribute to carcinogenesis and progression of the disease.

### Construction of histone acetylation patterns

**Fig. 2:**
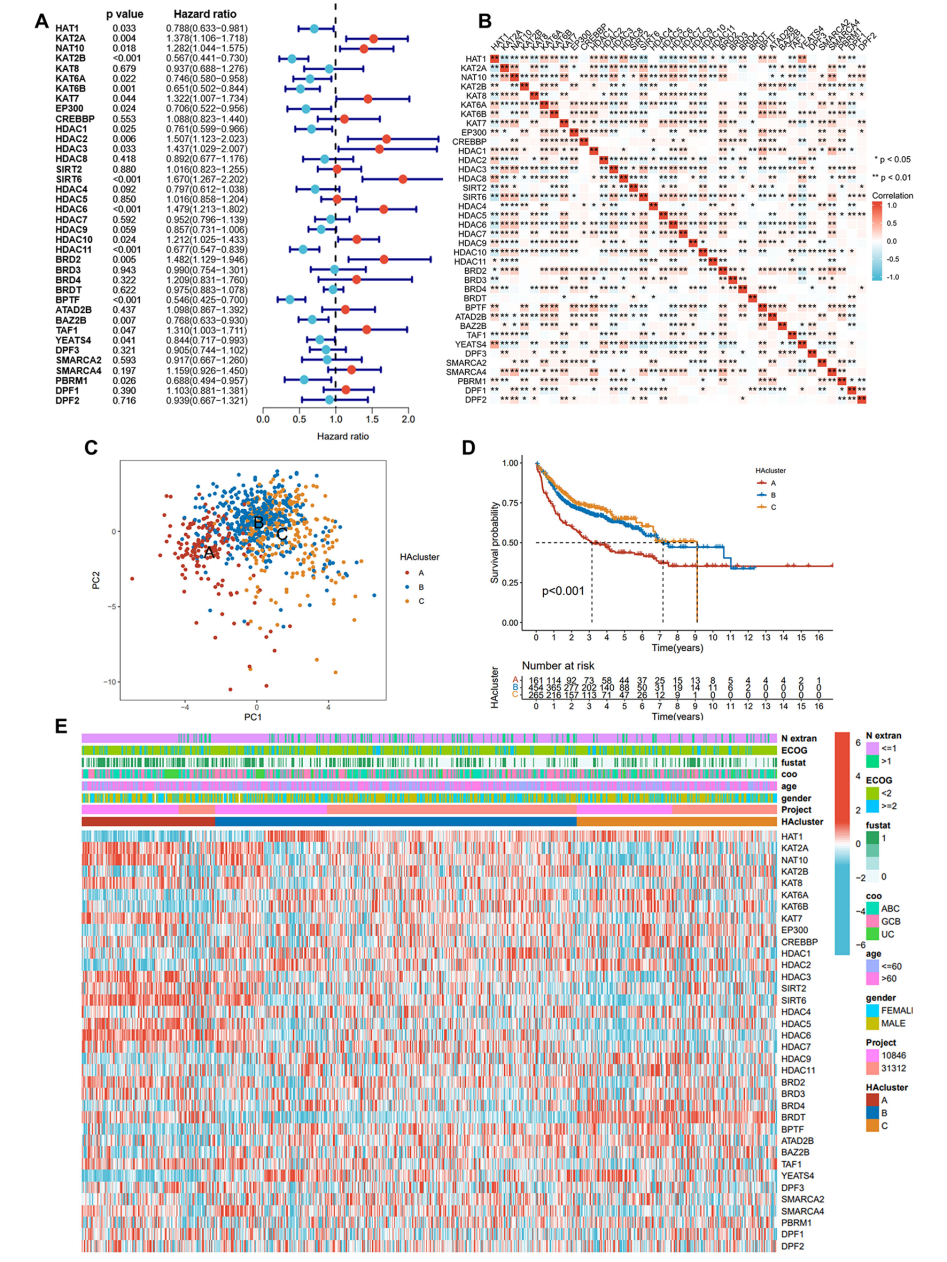
Construction of histone acetylation patterns. **(A)**: The univariable cox regression analyses of histone regulators for overall survival. **(B)**: Correlation between the HA regulators. **(C)**: PCA of the three histone acetylation modification patterns. **(D)**: Survival curves for three HAclusters in DLBCL. **(E)**: Unsupervised clustering of 38 HA regulators in DLBCL patients. HAcluster, survival status, ECOG, gender, N extra, and age are used to annotate DLBCL patients.

A univariate Cox regression model demonstrated the predictive significance of histone acetylation regulators (Fig. 2A and Table S2). The results showed that multiple regulators were risk factors for DLBCL, and some regulators were protective factors. An analysis of spearman correlations was conducted in order to describe the interactions among the regulator connections (Fig. 2B). According to the results, most of the regulators were positively correlated. A significant correlation was found between genes belonging to the same biological group (writers, erasers, and readers), and a positive correlation was also found between genes with opposite biological functions. The results suggest that there is extensive crosstalk among histone acetylation regulators, which collectively regulate histone acetylation modifications and influence DLBCL development. HA regulators were analyzed with unsupervised clustering to identify three patterns of histone acetylation. 884 DLBCL patient samples were grouped according to their gene expression, with 163 cases belonging to cluster A, 455 cases belonging to cluster B, and 266 cases belonging to cluster C (Fig. S1A-D). Significantly, PCA analysis revealed differences between the three different histone acetylation patterns (Fig. 2C). Prognostic analysis of the three subtypes of histone acetylation modifications revealed that HAcluster A had the worst prognosis (Fig. 2D). Additionally, the correlation between clinical factors and gene expression was analyzed. The results presented in Fig. 2E illustrated the correlation among three distinct histone acetylation patterns. Compared to the other two groups, HAcluster A showed a significantly higher incidence of activated B-cell–like (ABC) DLBCL. There is a well-established association between ABC DLBCL and a poor prognosis [7], indicating decreased survival observed among HAcluster A patients.

### Immune infiltration and biological process associated with histone acetylation patterns

**Fig. 3:**
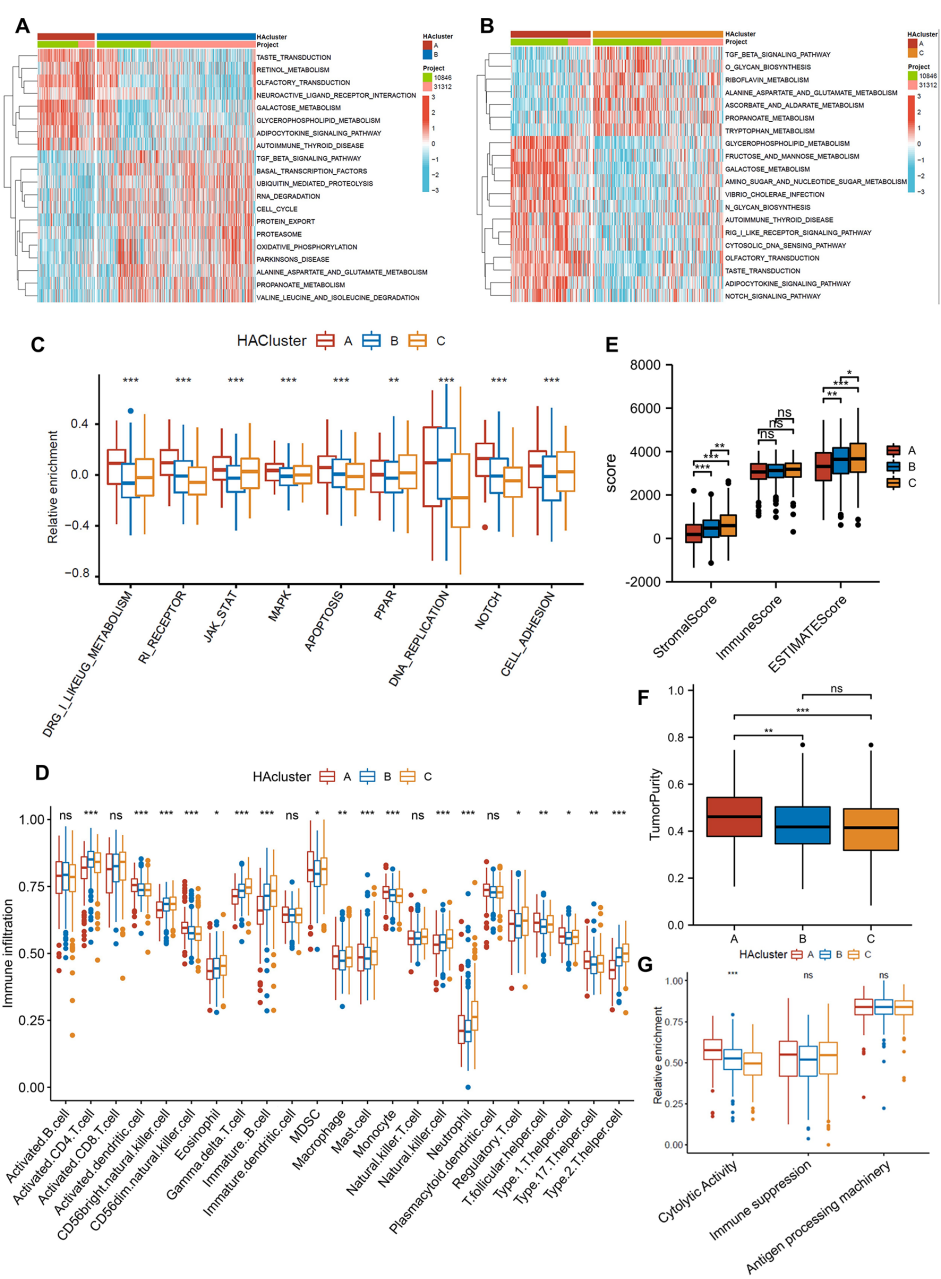
Histone acetylation patterns and their biological characteristics in DLBCL. **(A, B)**: Enrichment analysis of GSVA activated by biological pathways among different HAclusters. **(C)**: Malignant pathways that differ among the three different clusters. **(D)**: Expression of TME-infiltrated cells in the three HAclusters. **(E)**: ESTIMATEScore, ImmuneScore, and StromalScore among the three different HAclusters. **(F)**: TumorPurity among the three different HAclusters. **(G)**: Immune-related pathways differ among clusters.

In accordance with the KEGG gene set, GSVA enrichment analysis examined the differences in biological behavior among the three modification patterns (Fig. 3A-B, Fig. S1E). The results of GSVA analysis revealed that HAcluster A was enriched in several metabolically relevant pathways. Additionally, HAcluster A was enriched in pathways related to tumors, including the DRUG-METABOLISM/JAK-STAT/MAPK/NOTCH/PARP signaling pathway and apoptosis, in comparison to HAcluster B and HAcluster C (Fig. 3C). Subsequently, TME cell infiltration analysis showed substantial differences in histone acetylation patterns among the three groups, with HAcluster A exhibiting higher levels of activated dendritic cells and MDSCs and lower levels of nature killer cells compared to HAcluster B and HAcluster C (Fig. 3D). Based on RNA expression levels of DLBCL, the ESTIMATE algorithm was employed to calculate the stromal, immune, and estimated scores (Fig. 3E-F). The boxplots showed that HAcluster A had significantly lower StromalScore and ESTIMATEScore and had significantly higher tumor purity than the other two groups. This is consistent with the previous analysis. According to the previous research, we assessed the immunosuppressive, immune cytolytic, and antigen-processing effects (Fig. 3G) [37, 39]. The results showed that HAcluster A had the highest immunosuppression, which indicated that the tumor microenvironment of HAcluster A was significantly different.

### Construction of HA gene signature

**Fig. 4:**
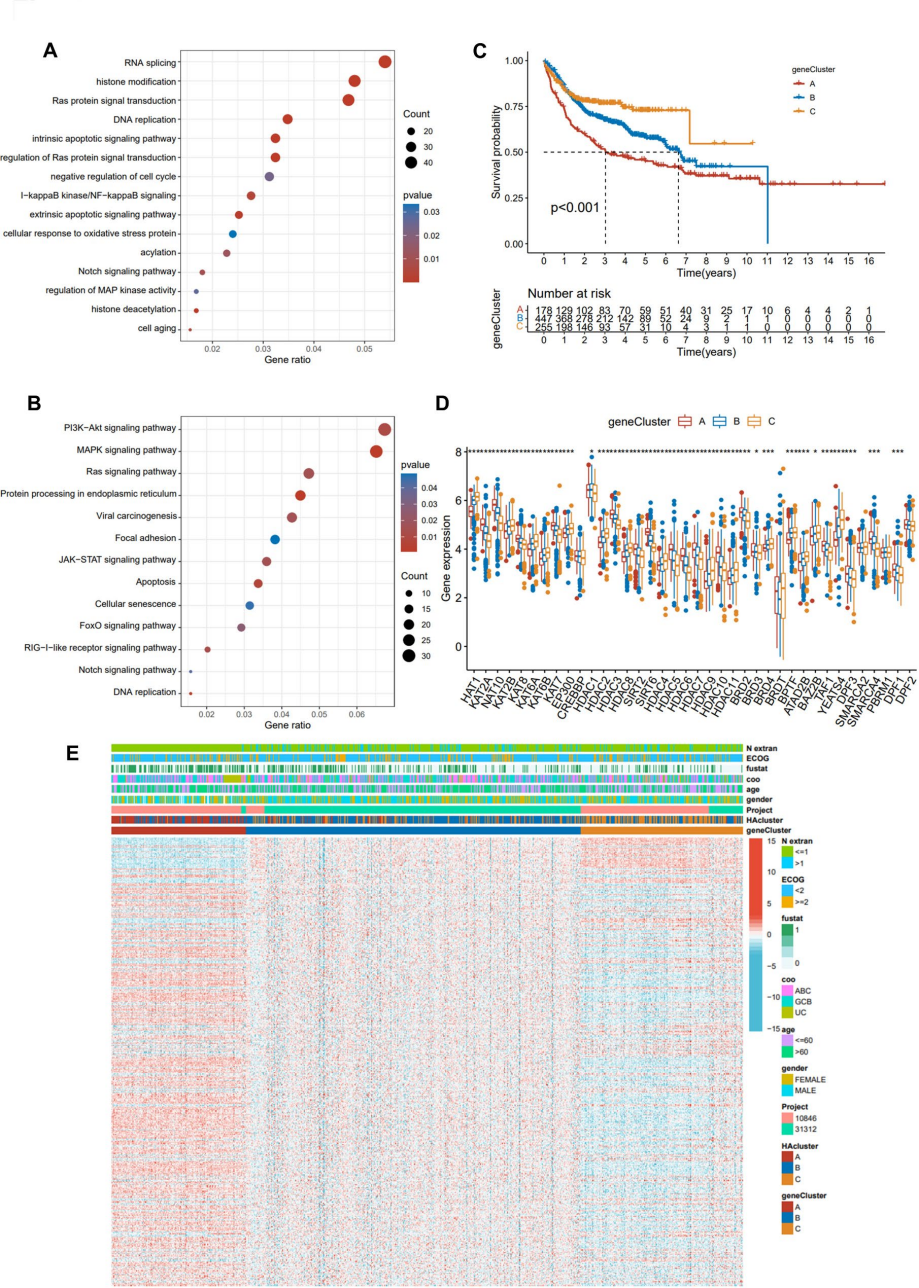
Construction of geneCluster and its prognostic significance. **(A, B)**: GO **(A)** and KEGG enrichment analysis **(B)** of HA-related genes. **(C)**: Kaplan-Meier curves of different geneClusters. **(D)**: Expression of 38 HA regulators in three geneClusters. **(E)**: Unsupervised clustering of HA regulators in the DLBCL cohort identified geneClusters A, B, and C. GeneClusters, HAclusters, survival status, N extra, age, COO, gender, and patient age were annotated.

For a better understanding of the differences among these three histone acetylation patterns, we identified 929 DEGs (Fig. S2A) significantly associated with patient survival in the previous cohort. This was followed by an unsupervised cluster analysis that classified patients into different genotypes (Fig. S2B-E). In the analysis of GO and KEGG enrichment of these DEGs, the main functions were found to be RNA splicing, histone modification, DNA replication, PI3K-AKT, MAPK, and Ras signaling (Fig. 4A-B). A model-based clustering analysis led to the identification of three different modification patterns, including 179 cases in geneCluster A, 450 cases in geneCluster B, and 255 cases in geneCluster C, respectively. In addition, a prognostic crossover could be observed for gene cluster A, which had a good prognosis (Fig. 4C). This suggests that these three different histone acetylation modification patterns are indeed present in DLBCL. As with previous HA modification patterns, significant differences were observed within HA geneClusters (Fig. 4D). Additionally, DEG expression varied significantly among the three HA geneClusters (Fig. 4E). GeneCluster A demonstrated the highest level of DEGs expression, followed by geneCluster B and C, which was also in line with patient survival.

### HAscore-related clinical features and TME immune activity

**Fig. 5:**
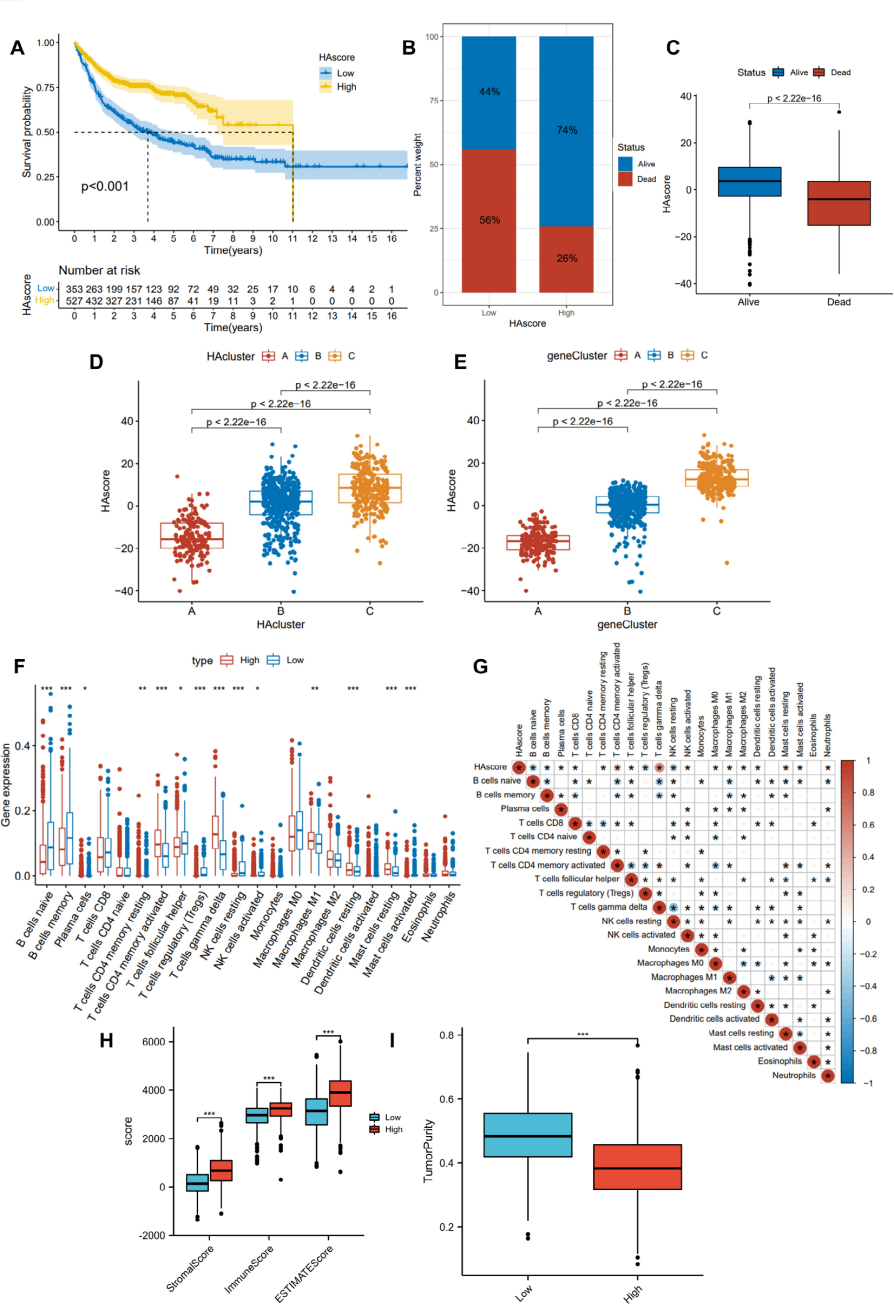
HA score groups with prognosis and immunological characteristics. **(A)** Kaplan–Meier curve of the patients with different HAscores cohort. **(B-C)**: The percentage of patients in low HAscore groups and high HAscore groups with differing survival outcomes. Differences between survival status groups in terms of HAscore. **(D)**: Differences between scores among three HAclusters in the DLBCL cohort. **(E)**: Difference in scores between three gene clusters in the DLBCL cohort. **(F-G)**: The differential and correlation between the HA score and TICs were analyzed using the CIBERSORT algorithm. **(H)**: The difference in TME scores between the groups according to their HAscore. **(I)**: TumorPurity between the HAscore groups.

A set of models of histone acetylation modification was developed based on individual differences and complexity of each DLBCL, called the HAscore, which can be used to quantify individual DLBCLs. In Fig. S3, the alluvial chart shows the change in the patient’s individual attributes. In HAcluster A, the majority of samples belonged to geneCluster A, which all had low HAscores. Also of note is that most of the patients in geneCluster B and C were in HAcluster B and C with high HA scores. Using the R package “survminer”, we calculated a cut-off value of -1.18397065. Patients were successfully classified into high and low groups according to their HAscores (Fig. 5A). Additionally, HAscore results indicated that a greater percentage of patients died in the low HAscore group, while surviving patients had significantly higher levels of HAscore than those who died (Fig. 5B-C). Based on these findings, high HAscore patients had a statistically significant survival advantage. Furthermore, Kruskal-Wallis analysis revealed significant differences in HAscores between HAclusters. A significantly lower mean HAscore was observed in HAcluster A and geneCluster A with poor prognoses (Fig. 5D-E). As a result, HAscore levels were strongly correlated with the survival benefits of patients.

For a more comprehensive understanding of the correlation between HAscores and the immune status of patients, we quantified different immune cell subsets and infiltration scores for each patient. Further analysis of the relationship between HAscore and TME revealed a positive correlation between immune cells and high HAscore levels. Based on the CIBERSORT algorithm, we confirmed the association of tumor-initiating cells (TICs) with HAscore (Fig. 5F-G). Furthermore, from the ESTIMATE algorithm, the low HAscore group had low expression in stromalscore, immunescore, and ESTIMATEscore (Fig. 5H). Consequently, the low HAscore group had a higher tumor purity, which is strongly correlated with poor prognosis (Fig. 5I). HAscore groups differed in terms of immune infiltration in this study. This suggests a regulatory role for histone acetylation regulators in the immune microenvironment of patients with DLBCL.

### KAT2A was expressed in high levels in DLBCL

**Fig. 6:**
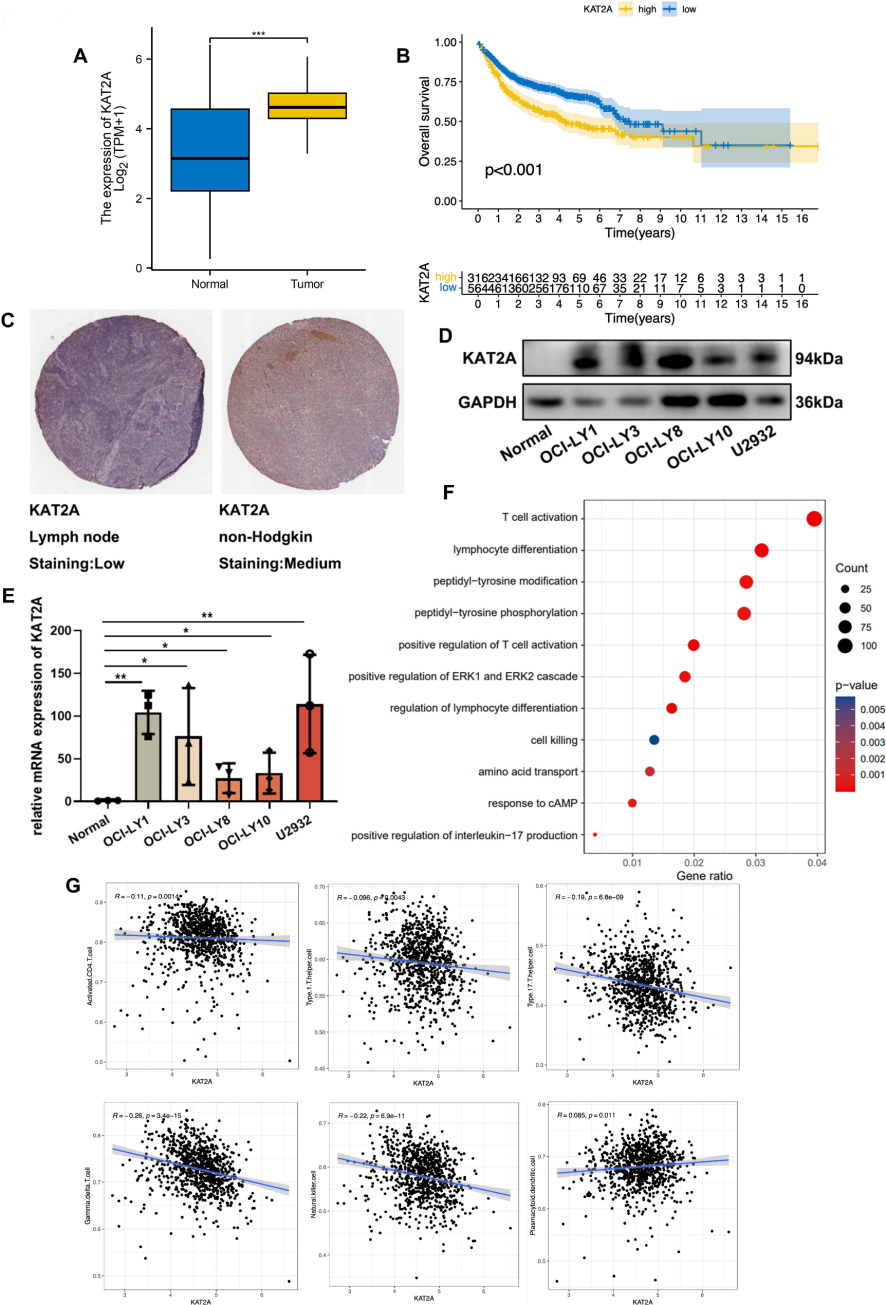
KAT2A was expressed in high levels in DLBCL. **(A)**: Expression of KAT2A in TCGA. **(B)**: Prognosis of KAT2A in GEO. **(C)**: Expression of KAT2A in NHL in public databases. **(D-E)**: KAT2A is highly expressed in DLBCL cell lines. **(F)**: GO analysis based on DEGs of KAT2A. **(G)**: A correlation of the abundance and correlation of TME infiltrating cells among different HAscore groups.

In light of the fact that KAT2A is closely related to several histone regulators, HAscore is able to predict the outcomes of DLBCL patients. In addition, we examined whether KAT2A might serve as a potential biomarker in the treatment of DLBCL. Based on the public database, KAT2A was overexpressed in DLBCL patients with poor prognoses (Fig. 6A-B). Similarly, KAT2A was found to be positive in non-Hodgkin’s lymphoma in HPA (Fig. 6C). Furthermore, we observed that KAT2A was mainly overexpressed in many DLBCL cell lines compared to CD19 + B cells in both protein and RNA levels (Fig. 6D-E). KAT2A was found to be associated with T cell activation as well as metabolic processes according to the GO analysis of DEGs (Fig. 6F). Moreover, we analyzed the relationship between KAT2A and TME and found a negative correlation with immune cells. It was found that there is a strong correlation between KAT2A and adaptive immune responses (such as activated CD4 T cells, Type 1 T helper cells, Type 17 T helper cells, Gamma delta T cells) and innate immune responses, such as plasmacytoid dendritic cells and natural killer T cells (Fig. 6G). In particular, low expression of KAT2A is closely associated with cells that perform antitumor functions.

#### Role of KAT2A in the progression of DLBCL

**Fig. 7:**
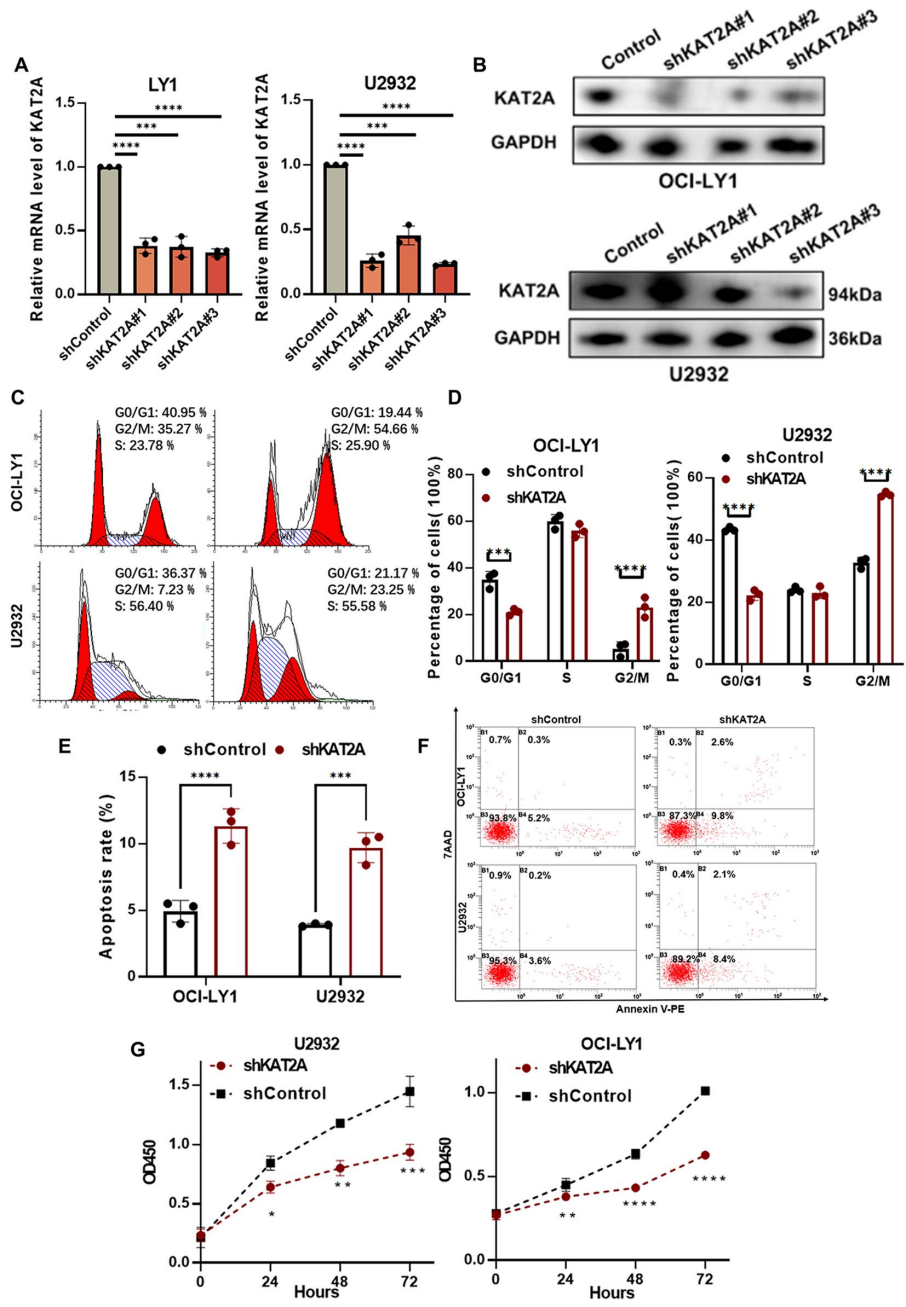
Inhibition of DLBCL progression by KAT2A knockdown. **(A-B)**: The knockdown effect of KAT2A was confirmed by RT-qPCR and protein blotting analyses. **(C-D)**: KAT2A knockdown arrests the cell cycle in the G2/M phase. **(E-F)**: KAT2A knockdown promoted cell apoptosis. **(G)**: KAT2A knockdown decreased cellular proliferative activity.

To elucidate the clinical significance of KAT2A in DLBCL, we knocked down KAT2A in two DLBCL cell lines (OCI-LY1 and U2932), resulting in stable KAT2A KD cell lines (Fig. 7A-B), of which shKAT2A#3 exhibited the highest efficacy. Further function investigations were performed based on shKAT2A#3 cell lines. By staining the cells with PI, we monitored the cell cycle. Compared with shControl cells, there was a significant increase in G2/M phase DLBCL cells after KAT2A was knocked down (Fig. 7C-D). We investigated the effect of KAT2A knockdown on apoptosis (Fig. 7E-F) and found that it significantly increased apoptosis rates. In addition, the stable KAT2A knockdown cells showed growth suppression as opposed to shControl cells (Fig. 7G). Therefore, KAT2A knockdown significantly inhibits the proliferation of the G2/M phase in DLBCL cell lines. As indicated by the findings of the study, KAT2A appeared to be capable of promoting DLBCL progression through its effects on proliferation, apoptosis, and cell cycle progression during the G2/M phase.

## Discussion

Histone acetylation is an epigenetic modification necessary for cancer biology. KAT7, for example, inhibits tumor cell proliferation and invasion by acetylating H4K5 at promoters of FOXO1 and FOXO3a genes [40], and HDAC5 loss increased H3K27-ac acetylation and circumvented oncogene-related cell cycle repression [41]. Considerable research has been conducted on individual histone acetylation regulators and their role in cancer; however, a comprehensive assessment of these regulators and their interactions remains lacking. Our research aims to develop a more complete understanding of the different histone acetylation patterns and their associated biological properties.

Based on a preliminary analysis of the expression of histone acetylation regulator genes, most genes were significantly different between normal and tumor samples with an association with prognosis. According to unsupervised clustering of 36 modulators, patients were categorized into three histone acetylation phenotypes. Survival analysis revealed three distinct patterns of histone acetylation modification associated with prognosis. To further characterize these different histone acetylation phenotypes, we determined DEGs among them. The HAscore model was developed based on these genes to assess histone acetylation phenotypes in individual patients quantitatively. All patients in HAcluster A have a low HAscore, associated with the poorest survival outcome. Moreover, we found a close relationship between most acetylation-related genes. One of these risk factors, KAT2A, has been found to be closely related to histone acetylation regulators.

In order to shed light on the mechanisms underlying the different prognoses of patients with different phenotypes, the biological characteristics of each pattern were examined. The results of GSVA analysis confirmed that HAcluster A showed enrichment in several metabolically relevant pathways. Tumorigenesis and tumor progression are supported by abnormal metabolic activity, which allows cells to obtain essential nutrients from the environment [42]. Moreover, we found that PARP, JAK-STAT, MAPK, NOTCH, and apoptosis were significantly activated in HAcluster A. Enrichment of these malignancy signaling pathways may be indicative of malignant progression, which may result in a poor prognosis [43–45].

A growing number of studies have suggested that TME components may contribute to cancer development [46–48]. An investigation into the relationship between histone acetylation modifications and TME cell infiltration was undertaken to to understand the antitumor immune response to DLBCL better. HAcluster A, with the lowest OS, exhibited a significantly higher level of MDSCs, which play a crucial role in immunosuppression [49]. MDSC is characterized by its ability to suppress immune cell function, including inhibition of T cell proliferation [50]. Each pattern is characterized by a different degree of TME infiltration, with immunosuppression characteristic of HAcluster A group. By analyzing histone acetylation patterns, we evaluated the potential therapeutic effects of HAscore based on differences in signaling pathways and tumor microenvironments between the different patterns. In the high-score group, higher levels of immune-activating cells were observed, including activated CD4 T cells and CD56 bright natural killer cells. CD4 + T cells have antitumor activity through the production of effector cytokines that activate CD8 + T cells [51]. By modulating DC and T cell responses, CD56 bright natural killer cells can positively influence the anticancer response [52]. This indicated that the HAscore contributed to further defining the microenvironment and thereby guiding more effective treatment.

Furthermore, our research revealed that KAT2A correlates with DLBCL prognosis and functions independently. An enzyme in the HAT family, lysine acetyltransferase 2 A (KAT2A), is involved in transcriptional activation through histone acetylation, histone succinylation, and recruitment of transcriptional coactivators [53]. Several studies have demonstrated that KAT2A functions as an epigenetic oncogene in several cancers [54, 55]. In previous studies, KAT2A has been shown to be a viable target for reducing the growth of acute myeloid leukemia by significantly promoting myeloid differentiation and apoptosis [56]. However, little was investigated between KAT2A and DLBCL. In this study, we utilized the bioinformatic methods to find that histone regulator KAT2A was a risk factor in DLBCL. The stable KAT2A KD cell lines were constructed for further study. Moreover, our research found that KAT2A deficiency could prohibit cell proliferation, promote cell apoptosis, and arrest cells in the G2/M phase. These findings suggest that inhibiting KAT2A may inhibit tumor growth.

In conclusion, our findings suggest that genetic signatures derived from histone acetylation regulators can be used to provide personalized survival assessments for patients newly diagnosed with DLBCL. It might provide clinicians with valuable information regarding treatment decisions, follow-up, and prognosis for their patients. There are some limitations to the study, despite its strengths. All the data we used were from public databases. It would be beneficial to collect more prospective real-world data to confirm its clinical utility. On the other hand, the specific mechanism of the effect of KAT2A in DLBCL still needs to be further explored.

### Electronic supplementary material

Below is the link to the electronic supplementary material.

Supplementary Material 1

Supplementary Material 2

Supplementary Material 3

## Data Availability

The datasets presented in this study can be found in online repositories. RNA sequencing data and CNV data were downloaded from the TCGA database (https://www.cancer.gov/) through the UCSC XENA website (https://xena.ucsc.edu/). The RNA data of normal control was downloaded from the Genotype-Tissue Expression (https://www.gtexportal.org/home/). And GSE10846 and GSE31312 were downloaded from the Gene Expression Omnibus (https://www.ncbi.nlm.nih.gov/geo/).

## Abbreviations

DLBCL: diffuse large B-cell lymphoma
TME: tumor microenvironment
NHL: non-Hodgkin’s lymphoma
DEGs: differentially expressed genes
HATs: histone acetyltransferases
HDACs: histone deacetylases
HDACi: histone deacetylases inhibition
GEO: Gene Expression Omnibus
TCGA: the Cancer Genome Atlas
TPM: transcripts per million
HA: histone acetylation
KEGG: Kyoto Encyclopedia of Genes and Genomes
GO: Gene Ontology
GSVA: Gene set variation analysis
OS: overall survival
SD: standard deviation
PCA: principal component analysis
TICs: tumor-initiating cells
ssGSEA: single-sample gene set enrichment analysis
CNVs: copy number variants
ABC DLBCL: activated B-cell–like DLBCL
KAT2A: lysine acetyltransferase 2 A

## References

[1] Zhang Y, Tan H, Daniels JD, Zandkarimi F, Liu H, Brown LM (2019). Imidazole Ketone Erastin induces ferroptosis and slows Tumor Growth in a mouse lymphoma model. Cell Chem Biol.

[2] Hartert KT, Wenzl K, Krull JE, Manske M, Sarangi V, Asmann Y (2021). Targeting of inflammatory pathways with R2CHOP in high-risk DLBCL. Leukemia.

[3] Melchardt T, Egle A, Greil R (2023). How I treat diffuse large B-cell lymphoma. ESMO Open.

[4] Miao Y, Medeiros LJ, Li Y, Li J, Young KH (2019). Genetic alterations and their clinical implications in DLBCL. Nat Reviews Clin Oncol.

[5] Yang J, Li Y, Zhang Y, Fang X, Chen N, Zhou X (2020). Sirt6 promotes tumorigenesis and drug resistance of diffuse large B-cell lymphoma by mediating PI3K/Akt signaling. J Experimental Clin cancer Research: CR.

[6] Liu Y, Barta SK (2019). Diffuse large B-cell lymphoma: 2019 update on diagnosis, risk stratification, and treatment. Am J Hematol.

[7] Chapuy B, Stewart C, Dunford AJ, Kim J, Kamburov A, Redd RA (2018). Molecular subtypes of diffuse large B cell lymphoma are associated with distinct pathogenic mechanisms and outcomes. Nat Med.

[8] Sehn LH, Herrera AF, Flowers CR, Kamdar MK, McMillan A, Hertzberg M (2020). Polatuzumab Vedotin in relapsed or refractory diffuse large B-Cell lymphoma. J Clin Oncology: Official J Am Soc Clin Oncol.

[9] Schuster SJ, Bishop MR, Tam CS, Waller EK, Borchmann P, McGuirk JP (2019). Tisagenlecleucel in adult relapsed or refractory diffuse large B-Cell lymphoma. N Engl J Med.

[10] Zhou X, Chen N, Xu H, Zhou X, Wang J, Fang X (2020). Regulation of Hippo-YAP signaling by insulin-like growth factor-1 receptor in the tumorigenesis of diffuse large B-cell lymphoma. J Hematol Oncol.

[11] Simithy J, Sidoli S, Yuan ZF, Coradin M, Bhanu NV, Marchione DM (2017). Characterization of histone acylations links chromatin modifications with metabolism. Nat Commun.

[12] Audia JE, Campbell RM (2016). Histone modifications and Cancer. Cold Spring Harb Perspect Biol.

[13] Sanchez GJ, Richmond PA, Bunker EN, Karman SS, Azofeifa J, Garnett AT (2018). Genome-wide dose-dependent inhibition of histone deacetylases studies reveal their roles in enhancer remodeling and suppression of oncogenic super-enhancers. Nucleic Acids Res.

[14] Razi S, Haghparast A, Chodari Khameneh S, Ebrahimi Sadrabadi A, Aziziyan F, Bakhtiyari M (2023). The role of tumor microenvironment on cancer stem cell fate in solid tumors. Cell Commun Signal.

[15] de Visser KE, Joyce JA (2023). The evolving tumor microenvironment: from cancer initiation to metastatic outgrowth. Cancer Cell.

[16] West AC, Johnstone RW (2014). New and emerging HDAC inhibitors for cancer treatment. J Clin Investig.

[17] Zhang P, Brinton LT, Williams K, Sher S, Orwick SA-O, Tzung-Huei L et al. Targeting DNA damage repair functions of two histone deacetylases, HDAC8 and SIRT6, sensitizes Acute myeloid leukemia to NAMPT Inhibition. (1557–3265 (Electronic)). 10.1158/1078-0432.CCR-20-3724 PMC8054771 33542077

[18] Verza FA, Das U, Fachin AL, Dimmock JR, Marins M. Roles of histone deacetylases and inhibitors in Anticancer Therapy. 2020;12(6):1664. 10.3390/cancers12061664 PMC7352721 32585896

[19] Mondello P, Tadros S, Teater M, Fontan L, Chang AY, Jain N (2020). Selective inhibition of HDAC3 targets synthetic vulnerabilities and activates Immune Surveillance in Lymphoma. Cancer Discov.

[20] Lasko LM, Jakob CG, Edalji RP, Qiu W, Montgomery D, Digiammarino EL (2017). Discovery of a selective catalytic p300/CBP inhibitor that targets lineage-specific tumours. Nature.

[21] Welti J, Sharp A, Brooks N, Yuan W, McNair C, Chand SN (2021). Targeting the p300/CBP Axis in Lethal prostate Cancer. Cancer Discov.

[22] Cai LY, Chen SJ, Xiao SH, Sun QJ, Ding CH, Zheng BN (2021). Targeting p300/CBP attenuates Hepatocellular Carcinoma Progression through Epigenetic Regulation of Metabolism. Cancer Res.

[23] Veazey KJ, Cheng D, Lin K, Villarreal OD, Gao G, Perez-Oquendo M (2020). CARM1 inhibition reduces histone acetyltransferase activity causing synthetic lethality in CREBBP/EP300-mutated lymphomas. Leukemia.

[24] Li X, Su X, Liu R, Pan Y, Fang J, Cao L (2021). HDAC inhibition potentiates anti-tumor activity of macrophages and enhances anti-PD-L1-mediated tumor suppression. Oncogene.

[25] Buglio D, Khaskhely NM, Voo KS, Martinez-Valdez H, Liu Y-J, Younes A (2011). HDAC11 plays an essential role in regulating OX40 ligand expression in Hodgkin lymphoma. Blood.

[26] Johnson WE, Li C, Rabinovic A (2007). Adjusting batch effects in microarray expression data using empirical Bayes methods. Biostatistics.

[27] Chen C, Grennan K, Badner J, Zhang D, Gershon E, Jin L (2011). Removing batch effects in analysis of expression microarray data: an evaluation of six batch adjustment methods. PLoS ONE.

[28] Xu Y, Liao W, Luo Q, Yang D, Pan M. Histone Acetylation Regulator-Mediated acetylation patterns define Tumor Malignant Pathways and Tumor Microenvironment in Hepatocellular Carcinoma. 2022;13. 10.3389/fimmu.2022.761046 PMC8821108 35145517

[29] Guo J, Wang Z, Wu J, Liu M, Li M, Sun Y (2019). Endothelial SIRT6 is vital to prevent hypertension and Associated Cardiorenal Injury through Targeting Nkx3.2-GATA5 signaling. Circul Res.

[30] Liu X, Tan Y, Zhang C, Zhang Y, Zhang L, Ren P (2016). NAT10 regulates p53 activation through acetylating p53 at K120 and ubiquitinating Mdm2. EMBO Rep.

[31] Wilkerson MD, Hayes DN (2010). ConsensusClusterPlus: a class discovery tool with confidence assessments and item tracking. Bioinformatics.

[32] Ritchie ME, Phipson B, Wu D, Hu Y, Law CW, Shi W (2015). Limma powers differential expression analyses for RNA-sequencing and microarray studies. Nucleic Acids Res.

[33] Kanehisa M, Goto S (2000). KEGG: kyoto encyclopedia of genes and genomes. Nucleic Acids Res.

[34] Kanehisa M (2019). Toward understanding the origin and evolution of cellular organisms. Protein Sci.

[35] Kanehisa M, Furumichi M, Sato Y, Kawashima M, Ishiguro-Watanabe M (2023). KEGG for taxonomy-based analysis of pathways and genomes. Nucleic Acids Res.

[36] Charoentong P, Finotello F, Angelova M, Mayer C, Efremova M, Rieder D (2017). Pan-cancer immunogenomic analyses reveal genotype-immunophenotype Relationships and Predictors of response to checkpoint blockade. Cell Rep.

[37] Sotiriou C, Wirapati P, Loi S, Harris A, Fox S, Smeds J (2006). Gene expression profiling in breast Cancer: understanding the molecular basis of histologic Grade to improve prognosis. JNCI: J Natl Cancer Inst.

[38] Mariathasan S, Turley SJ, Nickles D, Castiglioni A, Yuen K, Wang Y (2018). TGFβ attenuates tumour response to PD-L1 blockade by contributing to exclusion of T cells. Nature.

[39] Thorsson V, Gibbs DL, Brown SD, Wolf D, Bortone DS, Ou Yang T-H (2018). The Immune Landscape of Cancer. Immunity.

[40] Jie M, Wu Y, Gao M, Li X, Liu C, Ouyang Q (2020). CircMRPS35 suppresses gastric cancer progression via recruiting KAT7 to govern histone modification. Mol Cancer.

[41] Zhou Y, Jin X, Ma J, Ding D, Huang Z, Sheng H (2021). HDAC5 loss impairs RB repression of pro-oncogenic genes and confers CDK4/6 inhibitor resistance in Cancer. Cancer Res.

[42] Yu Z, Zhou X, Wang X (2022). Metabolic reprogramming in hematologic malignancies: advances and clinical perspectives. Cancer Res.

[43] Pradella D, Naro C, Sette C, Ghigna C (2017). EMT and stemness: flexible processes tuned by alternative splicing in development and cancer progression. Mol Cancer.

[44] Koushyar S, Powell AG, Vincan E, Phesse TJ. Targeting wnt signaling for the treatment of gastric Cancer. Int J Mol Sci. 2020;21(11). 10.3390/ijms21113927 PMC7311964 32486243

[45] Frontzek F, Staiger AM, Wullenkord R, Grau M, Zapukhlyak M, Kurz KS (2023). Molecular profiling of EBV associated diffuse large B-cell lymphoma. Leukemia.

[46] Liu Y, Zhou X, Wang X (2021). Targeting the tumor microenvironment in B-cell lymphoma: challenges and opportunities. J Hematol Oncol.

[47] Ennishi D, Hsi ED, Steidl C, Scott DW (2020). Toward a new molecular taxonomy of diffuse large B-cell lymphoma. Cancer Discov.

[48] Leivonen SK, Friman T, Autio M, Vaittinen S, Jensen AW, D’Amore F et al. Characterization and clinical impact of the tumor microenvironment in post-transplant aggressive B-cell lymphomas. Haematologica. 2023. 10.3324/haematol.2023.282831 PMC10620595 37259566

[49] Tian X, Shen H, Li Z, Wang T, Wang S (2019). Tumor-derived exosomes, myeloid-derived suppressor cells, and tumor microenvironment. J Hematol Oncol.

[50] Kim HJ, Ji YR, Lee YM (2022). Crosstalk between angiogenesis and immune regulation in the tumor microenvironment. Arch Pharm Res.

[51] Tay RE, Richardson EK, Toh HC (2021). Revisiting the role of CD4(+) T cells in cancer immunotherapy-new insights into old paradigms. Cancer Gene Ther.

[52] Sungur CM, Murphy WJ (2014). Positive and negative regulation by NK cells in cancer. Crit Rev Oncog.

[53] Wang Y, Chen W, Lian J, Zhang H, Yu B, Zhang M (2020). The lncRNA PVT1 regulates nasopharyngeal carcinoma cell proliferation via activating the KAT2A acetyltransferase and stabilizing HIF-1α. Cell Death Differ.

[54] Lu D, Song Y, Yu Y, Wang D, Liu B, Chen L (2021). KAT2A-mediated AR translocation into nucleus promotes abiraterone-resistance in castration-resistant prostate cancer. Cell Death Dis.

[55] Haque ME, Jakaria M, Akther M, Cho DY, Kim IS, Choi DK (2021). The GCN5: its biological functions and therapeutic potentials. Clin Sci (Lond).

[56] Tzelepis K, Koike-Yusa H, De Braekeleer E, Li Y, Metzakopian E, Dovey OM (2016). A CRISPR dropout screen identifies genetic vulnerabilities and therapeutic targets in Acute myeloid leukemia. Cell Rep.

