# Supplementary figures and images for: Histone regulator KAT2A acts as a potential biomarker related to tumor microenvironment and prognosis of diffuse large B cell lymphoma
Source: BMC Cancer. 2023 Oct 3;23:934. doi: 10.1186/s12885-023-11401-4 (PMC10546681; doi:10.1186/s12885-023-11401-4)

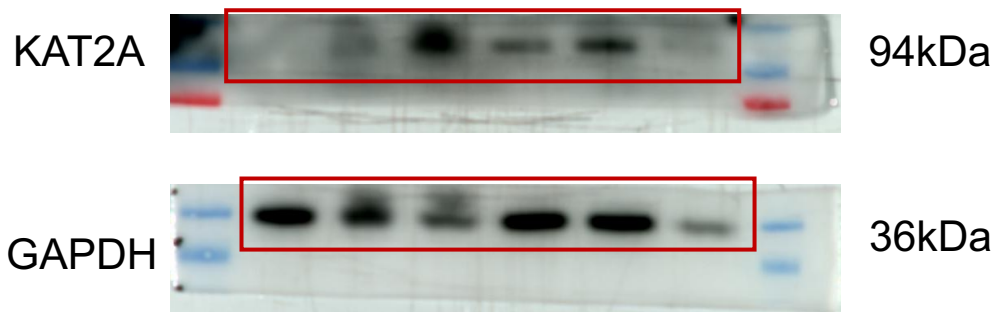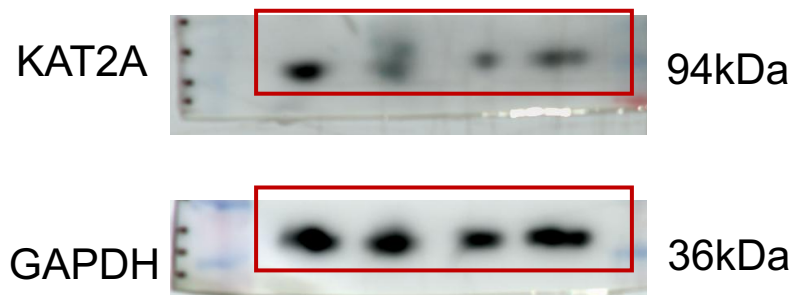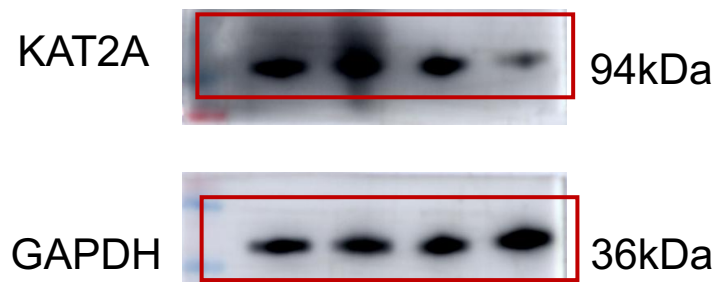

Supplement: Supplementary file 2 — Supplementary Material 2 [file 12885_2023_11401_MOESM2_ESM.pdf]
