# Supplementary material for: Histone regulator KAT2A acts as a potential biomarker related to tumor microenvironment and prognosis of diffuse large B cell lymphoma
Source: BMC Cancer. 2023 Oct 3;23:934. doi: 10.1186/s12885-023-11401-4 (PMC10546681; doi:10.1186/s12885-023-11401-4)
Supplement: Supplementary file 3 — Supplementary Material 3 [file 12885_2023_11401_MOESM3_ESM.docx]

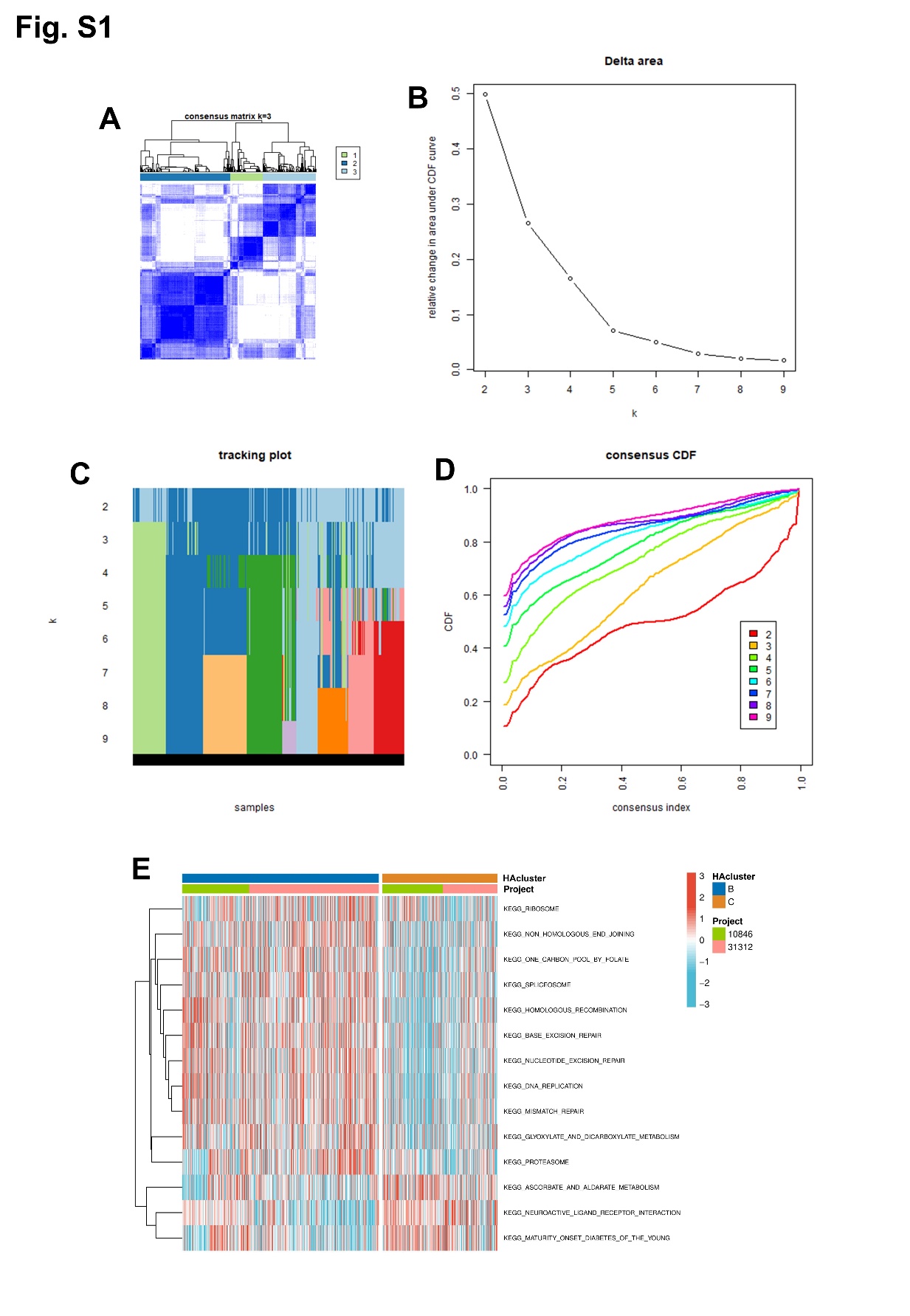


Fig. S1

Construction of histone acetylation regulators signatures. (A): Consensus matrices of the cohort for k =3. (B-D): Delta area curves (B), tracking plot (C), and consensus clustering cumulative distribution function (D) with k = 2 to 9. (E): Enrichment analysis of GSVA activated by biological pathways between HAcluster B and HAcluster C.


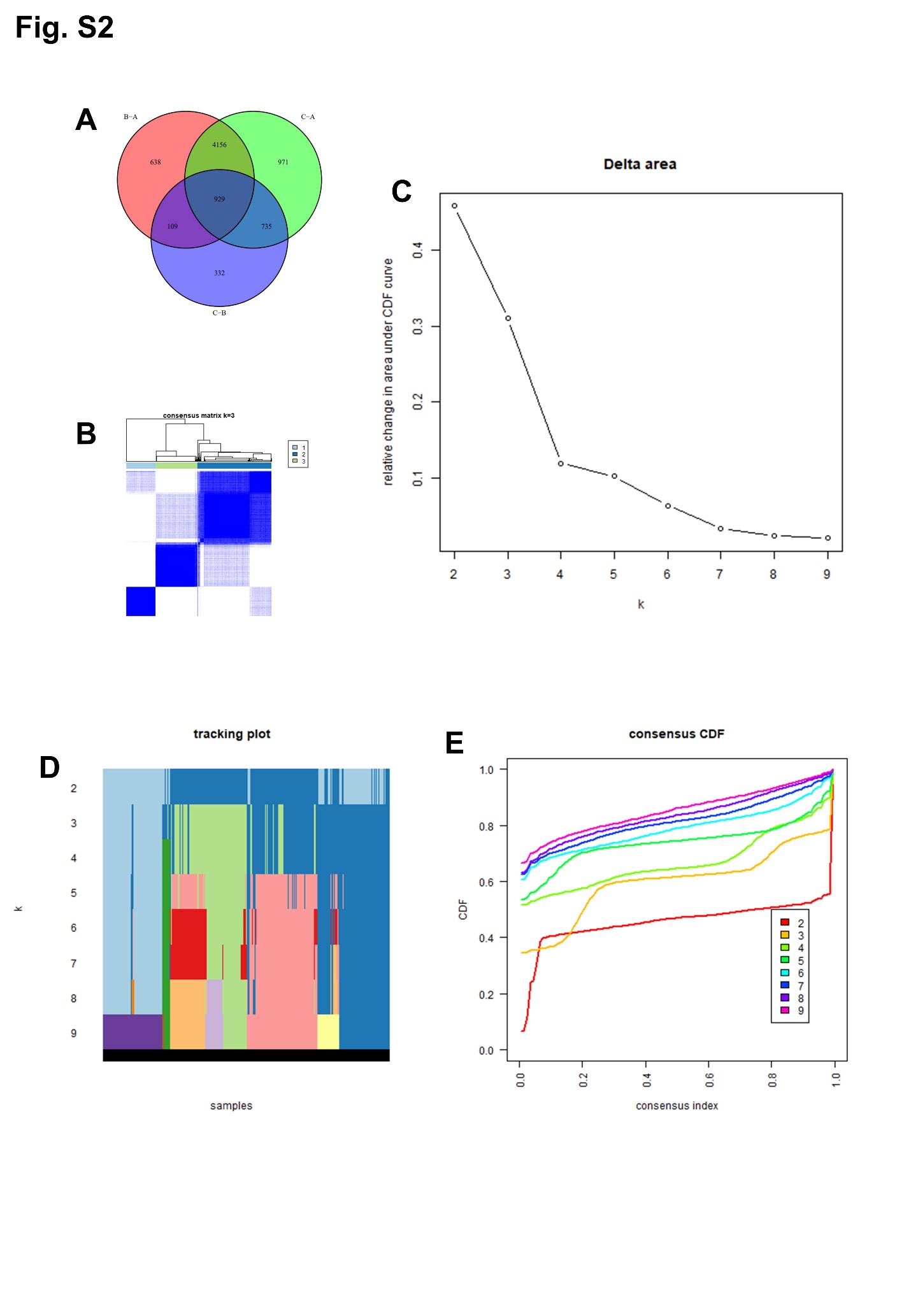


Fig. S2

Construction of HA-related DEGs signatures. (A) Venn diagram of 929 HA-related differentially expressed genes. (B): Consensus matrices of the cohort for k =3. (C-E): Delta area curves (C), tracking plot (D), and consensus clustering cumulative distribution function (E) with k = 2 to 9.


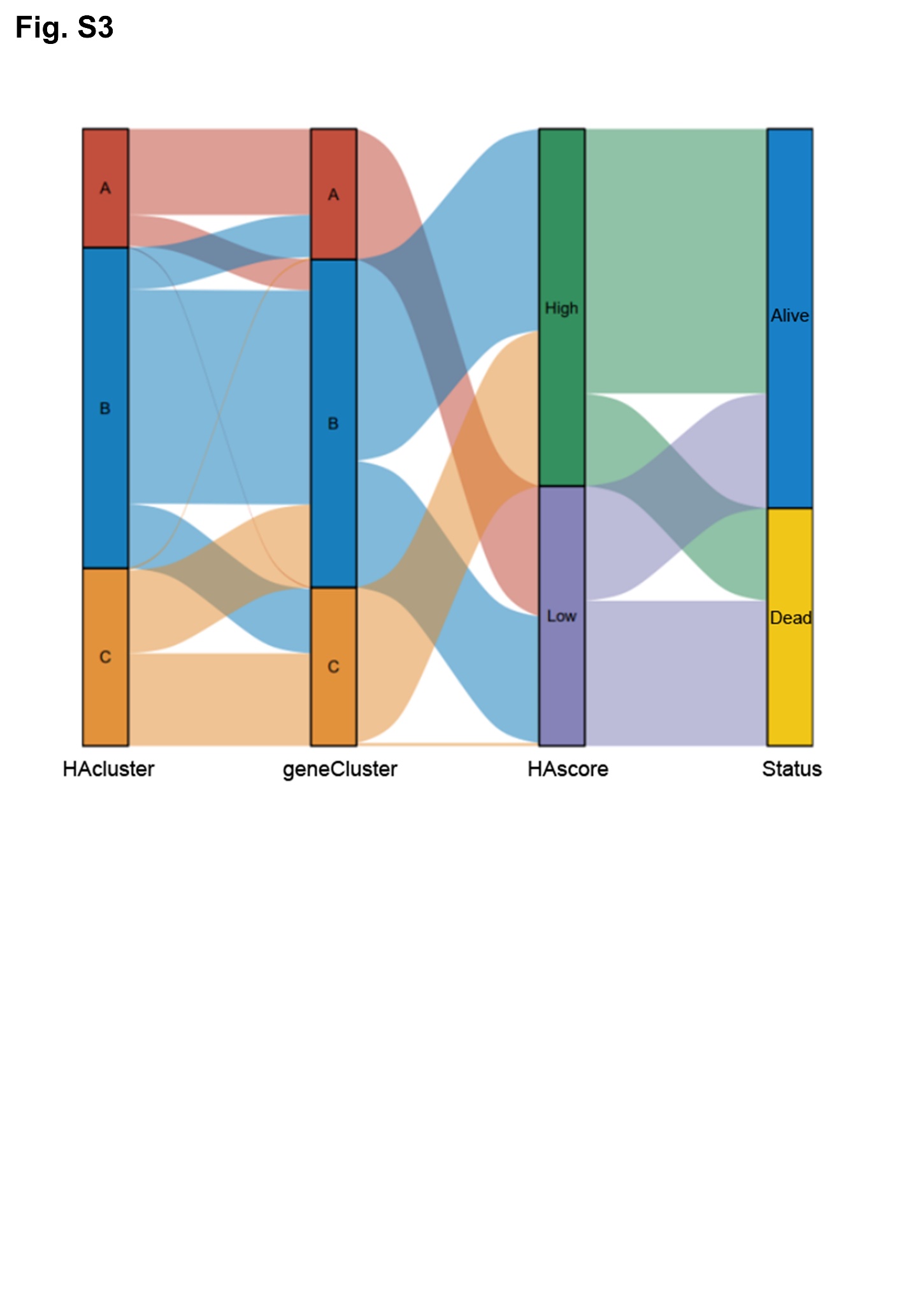


Fig. S3

The alluvial diagram displays the changes in the HA cluster, geneCluster, HAscore and survival outcome.
